# *Brevibacterium* Species Infections in Humans—A Narrative Review

**DOI:** 10.3390/microorganisms13051097

**Published:** 2025-05-09

**Authors:** Takis Panayiotou, Anastasia Vasilopoulou, Stella Baliou, Andreas G. Tsantes, Petros Ioannou

**Affiliations:** 1 School of Medicine, University of Crete, 71003 Heraklion, Greecestellabaliou@gmail.com (S.B.); 2 Laboratory of Hematology and Blood Bank Unit, “Attikon” University Hospital, School of Medicine, National and Kapodistrian University of Athens, 12462 Athens, Greece; andreas.tsantes@yahoo.com; 3 Microbiology Department, “Saint Savvas” Oncology Hospital, 11522 Athens, Greece

**Keywords:** *Brevibacterium*, bacteremia, infection, peritoneal dialysis-associated peritonitis, osteomyelitis, endophthalmitis

## Abstract

*Brevibacterium* species are Gram-positive, non-sporulating, coryneform, aerobic rods that are catalase positive and exhibit a distinctive transition from diptheroid to coccoid morphology during culture. Infections by these species are seldom identified. Objective: This narrative review aims to present all the reported cases of *Brevibacterium* spp. infections in humans, focusing on data about epidemiology, antimicrobial resistance, antimicrobial treatment, and mortality. A narrative review based on a literature search of PubMed/MedLine and Scopus databases was performed. In total, 41 studies providing data on 42 patients with *Brevibacterium* spp. infections were included in the present analysis. The median age was 48 years, while 57.5% were male. The presence of a central venous catheter and malignancy, and end-stage renal disease on peritoneal dialysis were the main predisposing factors. Bacteremia was the most common type of infection, with peritoneal dialysis-associated infections being the second most common. *B. casei* was the most commonly identified species. Microbial identification required the use of advanced molecular techniques, such as 16s rRNA sequencing or matrix-assisted laser desorption/ionization time of flight mass spectrometry in most cases. *Brevibacterium* spp. was highly resistant to the combination of trimethoprim with sulfamethoxazole, clindamycin, and common beta-lactams. The most commonly used antimicrobials were vancomycin and aminoglycosides. The mortality was about 10%. Clinicians and laboratory personnel should consider this pathogen in the differential diagnosis in patients with malignancy or peritoneal dialysis-associated peritonitis. Vancomycin should be used for empirical treatment and while antimicrobial susceptibility testing results are pending.

## 1. Introduction

*Brevibacterium* species are Gram-positive, non-sporulating, coryneform, aerobic rods that are catalase positive and exhibit a distinctive transition from diptheroid to coccoid morphology during culture [1]. Initially considered as environmental contaminants, *Brevibacterium* spp. are now recognized as opportunistic pathogens in humans, particularly in immunocompromised patients or those with indwelling medical devices [2,3]. The concept of opportunistic infection was introduced many decades ago after the notion that some microorganisms are more likely to cause infections in specific hosts. Thus, an opportunistic infection can be defined as a serious infection by a microorganism with limited pathogenic capacity in common circumstances, but it can cause serious diseases in the presence of predisposing factors such as some diseases or specific treatments [4]. An opportunistic pathogen may be able to cause infection in the absence of predisposing factors, but usually, the infection is more severe when occurring in patients with the predisposing factors [4].

The genus *Brevibacterium* includes various species, such as *B. casei*, *B. epidermidis*, *B. paucivorans*, *B. luteolum*, and *B. otitis*, but *B. casei* appears to be the most frequent species isolated from clinical specimens [2,5]. Historically, *Brevibacterium* spp. were associated with dairy products and human skin microbiota, contributing to cheese ripening and foot odor [6,7]. However, since the first reported case of bacteremia related to *Brevibacterium* spp. in 1969 in a patient with post-operative meningitis and prolonged fever, these organisms have been involved in a range of infections, including bacteremia, endocarditis, brain abscesses, peritonitis, osteomyelitis, and pericarditis [8,9,10,11]. Infections tend to arise most often among patients with central venous catheters, prosthetic devices, or underlying diseases such as malignancies or immunosuppression (e.g., patients with HIV infection) [12,13,14].

Diagnosis of *Brevibacterium* may be challenging as Gram stain usually shows Gram-positive rods like diphtheroids. Biochemical testing could further differentiate between other microorganisms [15]. Advanced molecular techniques such as either matrix-assisted laser desorption/ionization time of flight mass spectrometry (MALDI-TOF MS) or 16s-RNA sequencing are frequently used for identifying or confirming adequate pathogen identification. Other methods of identification can be the API Coryne gallery or a combination of microscopy, culture, biochemical tests, and chemical composition analysis [13,14,16].

Given the scarce data that are seldom reported mainly through case reports, the present study aimed to comprehensively review all the available information of all the types of human infections caused by this species in the literature and to assess the clinical data, microbiology, treatment, and outcomes.

## 2. Materials and Methods

### 2.1. Search Strategy and the Inclusion and Exclusion Criteria

This review aims to present all the data on *Brevibacterium* species infections in humans that have been published in the literature. The primary aim of the study was to present the data on patients’ demographics, clinical characteristics, and mortality. The secondary aims were to present the data regarding the infection site, the clinical presentation, the microbiological characteristics regarding species, identification, and antimicrobial resistance, and the treatment provided for the infection. For this narrative review, the PubMed/Medline and Scopus databases were searched until 13 February 2025. Data were extracted using a predefined template. The following keywords were used for the search strategy: “*Brevibacterium*” AND “infection”. Studies providing original data, such as case series, case reports, and cohort studies providing information into the epidemiology and clinical outcomes of *Brevibacterium* spp. infections in humans were included. Studies not in the English language, reviews, and systematic reviews were excluded. Studies in animals, and articles without full-text access, were also excluded from the analysis. Additionally, cases of colonization by *Brevibacterium* species were excluded from the analysis. The references of all the included articles were examined to identify any studies potentially missed in the initial search.

### 2.2. Data Extraction and Definitions

The data extracted from each included study were the publication year, article type, country of origin, patient demographics (age, sex), relevant medical history, details of infection, and the key clinical characteristics, such as the specific infection site, complications, as well as microbiological characteristics, such as the identified pathogen, antibiotic susceptibilities, and finally, the treatment used and outcome (survival or mortality). The relationship between mortality and the initial infection was documented according to each study’s authors.

### 2.3. Statistical Analysis

Data are presented as numbers (%) for categorical variables and median (interquartile range, IQR) for continuous variables. Continuous variables were compared using the Mann–Whitney U-test for non-normally distributed variables or the *t*-test for normally distributed variables. All the tests were two-tailed, and a *p*-value equal to or lower than 0.05 was considered significant.

## 3. Results

### 3.1. Included Studies’ Characteristics

**Figure 1:**
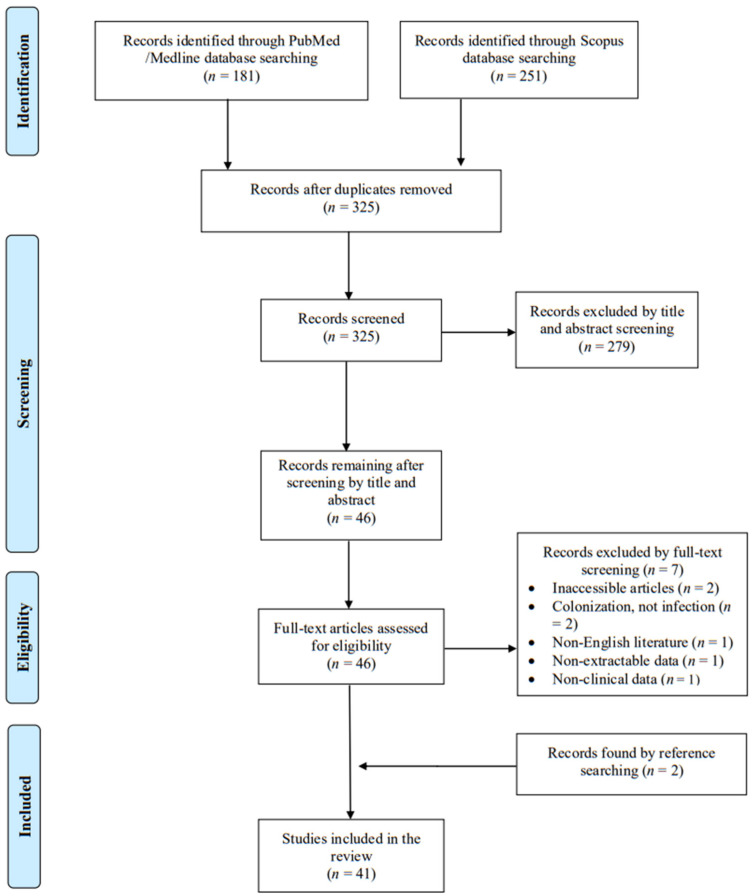
Trial flow of this narrative review.

A total of 325 articles were screened from the PubMed and Scopus databases. Eventually, after duplicate removal, record screening, and applying the snowball procedure, only 41 articles met the inclusion criteria and were selected for analysis [2,3,8,9,10,12,13,14,16,17,18,19,20,21,22,23,24,25,26,27,28,29,30,31,32,33,34,35,36,37,38,39,40,41,42,43,44,45,46,47,48]. These studies presented data on 42 patients. A flow diagram of the selection process is illustrated in Figure 1. Among the included cases, 21 were diagnosed in Europe (50%), 12 in Asia (28.6%), and 9 in North and South America (21.4%). Among the 41 articles that were eventually included, 39 (95.1%) were case reports. Table 1 shows the characteristics of the included studies in the present review. The data sheet can be found in File S1.

### 3.2. Epidemiology of Brevibacterium spp. Infections

The median age of patients with *Brevibaterium* spp. infections was 48 years, with a range of 0 to 94 years, while 57.5% (23 out of 40 patients with available data) were male. Regarding patients’ medical history and predisposing risk factors, 17 out of 41 (41.5%) had a central venous catheter, 10 out of 40 (25%) had an active malignancy which was hematologic in 6 patients (15% of all patients), 7 out of 40 (17.5%) had end-stage kidney disease on peritoneal dialysis, 6 out of 36 patients (16.7%) had had surgery in the preceding three months, 3 out of 40 patients (7.5%) were people living with the human immunodeficiency virus (PLWHIV), and 2 out of 41 patients (4.9%) had had organ transplantation. The demographic and clinical characteristics of patients with infections by *Brevibacterium* spp. are shown in Table 2.

### 3.3. Microbiology and Antimicrobial Resistance of Brevibacterium spp. Infections

*Brevibacterium* spp. was isolated in the blood of twenty-four patients (59.5%), from peritoneal fluid in seven (16.7%), from pus or tissue cultures in five (11.9%), from vitreous fluid in two (4.8%), from cerebrospinal fluid in two (4.8%), and from pericardial fluid, or ventriculoperitoneal shunt valve culture in one (2.4%) each. *B. casei* was the identified species in twenty patients (47.6%), *B. epidermidis* and *B. otitidis* were identified in three (7.1%) each, and *B. iadinum*, *B. paicivorans*, *B. luteolum*, and *B. sanguinis* were identified in one patient (2.4%) each. In ten patients (23.8%), the species was not reported. In 14 patients (33.3%), identification was performed with matrix-assisted laser desorption/ionization time of flight mass spectrometry (MALDI-TOF MS), and in 13 patients (31%), 16s-rRNA sequencing was used for pathogen identification. The API Coryne gallery was used for identification in 10 patients (23.8%), and a combination of microscopy, culture, biochemical test, and chemical composition analysis was used for identification in 1 patient (2.4%). The means of pathogen identification were not mentioned in eight patients (19%). The antimicrobial resistance of *Brevibacterium* spp. is shown in Table 3. In 2 out of 42 patients (4.8%), the infection was polymicrobial.

### 3.4. Clinical Presentation of Brevibacterium spp. Infections

The most common type of *Brevibacterium* spp. infections were those of the bloodstream in 24 patients (57.1%). Peritoneal dialysis-associated peritonitis was diagnosed in seven (4.8%), central nervous system infections in four (9.5%), osteoarticular infections in three (7.1%), infective endocarditis in three (7.1%), skin and soft tissue infections in two (4.8%), endophthalmitis in two (4.8%), and VP shunt-associated infections in one (2.4%). The symptoms’ duration ranged from one day to more than 60 days.

### 3.5. Treatment and Outcome of Brevibacterium Infections

The treatment of patients with *Brevibacterium* spp. infections is shown in detail in Table 1 and is also summarized in Table 2. Based on the available data, vancomycin was the most frequently administered antimicrobial used in twenty-three out of forty patients with available data (57.5%), followed by cephalosporins in nine (22.5%), aminoglycosides in eight (20%), quinolones in six (15%), teicoplanin in four (10%), carbapenem and aminopenicillins in three (7.5%) each, antipseudomonal penicillin, macrolides, and daptomycin in two (5%) each, and antistaphylococcal penicillin, linezolid, rifampicin, and tetracyclins in one (2.5%) each. Surgical interventions were applied in combination with antimicrobial treatment in 10 out of 41 patients (24.4%). The median treatment duration for survivors was 19.5 days. The overall mortality rate was estimated at 10.3% (4 out of 39 patients with available data), with the mortality directly associated with the *Brevibacterium* spp. infection being 7.7% (3 patients).

### 3.6. Bacteremia Due to Brevibacterium

Bacteremia was diagnosed in 24 patients (57.1%). Among them, 11 (50% among 22 with available data) were male and the median age was 46 years. Among these patients, 73.9% (17 out of 23 with available data) had a central venous catheter, 39.1% (9) had malingnancy that was hematological in 66.7% of them, and 27.3% (6 out of 22) had neutropenia. Fever was present in 91.7% (22 out of 24 patients) and sepsis was present in 34.8% (8 out of 23). Vancomycin and aminoglycosides were the most commonly used antimicrobials. The overall mortality was 13% (3 out of 23 patients).

### 3.7. Peritoneal Dialysis-Associated Peritonitis Due to Brevibacterium

Peritoneal dialysis-associated peritonitis was diagnosed in seven patients. The median age in this patient group was 63 years old, and four patients were male (57.1%). The diagnosis was made using peritoneal dialysis fluid in all patients. Fever was present in 57.1% (four out of seven patients) but no patient had sepsis. The median treatment duration was 24.5 days. The most commonly used antimicrobial agents were vancomycin cephalosporins, and aminoglycosides. No patient died due to this infection.

### 3.8. Characteristics of Patients with Brevibacterium spp. in Regard to Survival

Table 4 shows a comparison of the characteristics of patients with *Brevibacterium* spp. who lived with those who died. A statistical analysis by directly comparing patients who survived with those who died was not pursued due to the small number of patients, especially those who died. However, patients who died had a higher age.

## 4. Discussion

The present narrative review summarizes the characteristics of infections by *Brevibacterium* spp. in humans by gathering all the published studies in the literature that provide the relevant clinical and microbiological data. The most common types of infections were bacteremia and peritoneal dialysis-associated peritonitis. Antimicrobial resistance to the combination of trimethoprim with sulfamethoxazole, and clindamycin was very common, while the resistance to first-line beta lactams was also common, while vancomycin, tetracyclines, and cabapenems were active in the vast majority of cases. Vancomycin, cephalosporins, and aminoglycosides were the most commonly used antimicrobials for treating these infections. The mortality from infections by *Brevibacterium* spp. was relatively low.

Several reports of infections by *Brevibacterium* spp. have been published in the last decades. Given the rarity of these bacteria as causes of human infections and the possibility for their misidentification when only common microbiological techniques relying on morphology and biochemical assays are used, more advanced techniques such as 16s rRNA sequencing and MALDI-TOF MS may be required for adequate identification in patients with such rare bacteria [49,50,51]. Indeed, in the present review, MALDI-TOF MS and 16s rRNA sequencing were used in more than half of the patients.

Among the patients’ medical history, the most commonly reported condition was the presence of a central venous catheter, the history of a malignancy, most commonly hematological, and the history of end-stage kidney disease on peritoneal dialysis. The first two conditions were associated with infections of the bloodstream. Indeed, among the different clinical presentations, bacteremia was the most common presentation, with peritoneal dialysis-associated peritonitis being the second more common. The presence of malignancy, either hematological or solid is a well-known risk factor for infection. These patients are usually treated with chemotherapy that leads to immunosuppression, making these patients more susceptible to infection, and severe complications, such as sepsis [52,53,54]. Patients with hematological malignancy are at a particularly high risk of infections, due to the neutropenia seen in patients treated with myeloablative chemotherapy and due to the underlying disease. Additionally, some of these people have hypogammaglobulinemia, while the mucosal damage due to the treatment provided and the common use of intravascular devices also add to the high infection risk [53,54]. The presence of these other risk factors could not have been evaluated via the present review since the information provided by the case reports included herein is brief; thus, the presence of mucosal damage and hypogammaglobulinemia could not have been recorded during data extraction. However, neutropenia was identified in many patients, underlining the high risk that these patients have, specifically for bacteremia when treated for the malignancy. In a study that did not provide adequate data to allow inclusion in the present review, Shweta et al. identified 48 isolates from 45 unique patients, with about 31% of those having malignancy and 20% being on chemotherapy at the time of diagnosis [55]. Moreover, about 15% had received stem cell or solid organ transplantation. These are confirmatory of the results of the present review.

Peritoneal dialysis was also a common risk factor among the patients with *Brevibacterium* spp. infection and was associated only with peritoneal dialysis-associated peritonitis by these pathogens. Most cases of peritoneal dialysis-associated peritonitis are caused by bacteria. The majority of cases are due to Gram-positive bacteria, and more specifically, staphylococci, streptococci, enterococci, and corynebacterial, while Gram-negative bacteria are the cause in up to 35%. In up to 25% of cases, the infection was polymicrobial [56,57,58,59]. However, several rare pathogens are identified nowadays and are reported in the literature given the better microbiological identification techniques that are available [60,61,62,63].

In the present review, beyond bacteremia, and peritoneal dialysis-associated peritonitis, some cases of infective endocarditis, central nervous system infection, skin and soft tissue infection, osteoarticular infection, prosthetic material in the central nervous system, and endophthalmitis were also identified. In the study by Shweta et al., 70% of the infections were bacteremias, while the other types were not mentioned [55]. However, the rates may vary depending on the type of hospital, while the fact that not all cases of *Brevibacterium* spp. may have been reported in the literature may have affected the types of infection depicted in the present review.

In the present study, the antimicrobial resistance to the combination of trimethoprim with sulfamethoxazole, clindamycin, and commonly used beta-lactams was higher than 50%. The antimicrobial resistance to carbapenems, tetracyclines, aminoglycosides, and vancoymycin was low. These results are similar to those published by Shweta et al., where the antimicrobial resistance to common beta-lactams and susceptibility to vancomycin were also shown [55]. Thus, vancomycin can be used for the empirical treatment of these infections while antimicrobial susceptibility results are pending. Indeed, in most of the patients included in the analysis of the present review, vancomycin was the most commonly used antimicrobial for treating these infections by *Brevibacterium* spp. Even though a narrative review with such a low number of included patients cannot provide strong recommendations, given the inability to draw firm conclusions by other means and the lack of international guidelines for the empirical treatment of these infections, it is reasonable to consider vancomycin, for empirical treatment when *Brevibacterium* spp. is identified and until the results of the antimicrobial susceptibility testing are available.

The mortality of *Brevibacterium* infections was about 10%. Importantly, three out of four patients who died had bacteremia. However, even though in this review, an attempt to compare patients who survived with those who died was performed, the small number of patients who died precluded statistical analysis. Thus, future studies should focus on prospectively evaluating the pathophysiology, as well as the clinical and microbiological characteristics of patients with *Brevibacterium* spp. infections to allow more adequate identification of those characteristics that could allow adequate pathogen identification, and the appropriate empirical treatment of patients with such infections.

This study has some notable limitations. First of all, the studies included in the present review may not be representative of the infections by *Brevibacteriun* spp. due to the problems associated with the identification of the pathogen and the reasonable possibility that not all cases of *Brevibacterium* spp. infections have been published. Additionally, some studies may have been missed during the screening process. Moreover, this review identified only a small number of studies, mostly case reports, that carry a specific risk of bias, providing data for only a small number of patients, thus limiting the credibility of the conclusions of the study. Finally, even though in the present review, we tried to evaluate the outcome of mortality, the heterogeneity in reporting the mortality in the case reports that provided data in the present study made it impossible to evaluate it at a specific timepoint, even though this outcome probably reflects the hospital mortality in most cases.

## 5. Conclusions

The present review provides important information about *Brevibacterium* spp. infections in humans. *B. casei* was the most commonly identified species, while the most common infections were those of the bloodstream and peritoneal dialysis-associated infections. MALDI-TOF MS or 16s rRNA sequencing can aid in microbial identification. *Brevibacterium* spp. had significant antimicrobial resistance to the combination of trimethoprim with sulfamethoxazole, clindamycin, and common beta lactams, while the antimicrobial resistance to vancomycin, carbapenems, tetracyclines, and aminoglycosides was very low. *Brevibacterium* spp. infections should be empirically treated with vancomycin until antimicrobial susceptibility tests are pending, especially in the case of bacteremia, that may carry a higher risk of mortality.

## Figures and Tables

**Table 1:** Characteristics of all included studies.

Author, Year	Number of Patients	Sex	Age (Years)	Site of Infection	Antimicrobial Treatment	Mortality (%)
McCaughey et al., 1991 [10]	1	Male	40	BSI	Macrolide	0
Neumeister et al., 1993 [17]	1	Male	1 month	Osteoarticular infection	Antistaphylococcal penicillin, cephalosporin	0
Lina et al., 1994 [18]	1	Male	19	BSI	Teicoplanin, aminoglycoside	0
Reinert et al., 1995 [19]	1	Male	25	BSI	Piperacillin, teicoplanin, aminoglycoside	0
Kaukoranta-Tolvanen et al., 1995 [20]	1	Female	46	BSI	Cephalosporin	0
Antoniou et al., 1997 [21]	1	Female	69	PD-associated peritonitis	Quinolone	0
Castagnola et al., 1997 [22]	1	NR	NR (child)	BSI	NR	NR
Wauters et al., 2000 [23]	1	Female	73	PD-associated peritonitis	Cephalosporin, aminoglycoside	0
Brazzola et al., 2000 [12]	1	Female	18	BSI	Quinolone	0
Dass et al., 2002 [16]	1	Female	68	BSI, IE	Vancomycin, macrolide, aminoglycoside	0
Öǧünç et al., 2002 [24]	1	NR	60	BSI	Vancomycin	0
Janda et al., 2003 [25]	1	Male	34	BSI	Vancomycin	0
Beukinga et al., 2004 [13]	2	1 female and 1 male	31, 43	2 BSIs	2 vancomycin	1 (50%)
Cannon et al., 2005 [8]	1	Male	78	Pericarditis	Vancomycin	1
Ulrich et al., 2006 [26]	1	Female	62	BSI	Vancomycin, quinolone	0
Roux et al., 2009 [27]	1	Male	78	Osteoarticular infection	NR	NR
Kumar et al., 2011 [9]	1	Male	31	CNS infection	Aminopenicillin, cephalosporin	0
Manetos et al., 2011 [28]	1	Male	52	BSI, IE, SSTI	Vancomycin, quinolone, aminoglycoside	0
Poesen et al., 2012 [29]	1	Male	37	PD-associated peritonitis	Vancomycin	0
Choi et al., 2012 [30]	1	Male	52	PD-associated peritonitis	Cephalosporin	0
Banu et al., 2013 [31]	1	Male	12	Endophthalmitis	Cephalosporin, vancomycin, aminoglycoside	0
Talento et al., 2013 [32]	1	Female	54	SSTI	Vancomycin, daptomycin	0
Mohammed et al., 2014 [33]	1	Female	33	PD-associated peritonitis	Vancomycin	0
Bal et al., 2015 [34]	1	Male	6	BSI	Vancomycin	0
Vecten et al., 2017 [35]	1	Male	4	BSI	Quinolone	0
Piccinelli et al., 2018 [36]	1	Female	49	BSI	Piperacillin & tazobactam, vancomycin, tigecycline	0
Magi et al., 2018 [37]	1	Female	48	BSI	Teicoplanin, linezolid	0
Asai et al., 2019 [3]	1	Female	94	BSI	Carbapenem, teicoplanin	1
Joshi et al., 2020 [38]	1	Male	6	BSI	Carbapenem, aminoglycoside	0
Olate-Pérez et al., 2020 [39]	1	Male	49	Endophthalmitis	Quinolone	0
Kimura et al., 2021 [40]	1	Male	72	PD-associated peritonitis	Cephalosporin, vancomycin, aminoglycoside	NR
Ovsthus et al., 2021 [41]	1	Male	0 (neonate)	CNS shunt infection	Vancomycin, rifampicin	0
Eidensohn et al., 2021 [2]	1	Male	40	Osteoarticular infection	Aminopenicillin, cephalosporin, vancomycin, daptomycin	0
Hossain et al., 2021 [42]	1	Male	71	BSI	Aminopenicillin & inhibitor	0
Benson et al., 2021 [43]	1	Female	85	BSI	Vancomycin	0
Ochi et al., 2021 [14]	1	Female	8	BSI	Carbapenem, vancomycin	0
Munshi et al., 2022 [44]	1	Male	64	BSI	Vancomycin	0
Roy et al., 2022 [45]	1	Male	63	PD-associated peritonitis	Vancomycin	0
Okoli et al., 2023 [46]	1	Female	43	VP shunt-associated infection	Cephalosporin	0
Aydemır et al., 2023 [47]	1	Female	54	BSI, IE	Vancomycin	1
Nguyen et al., 2025 [48]	1	Female	60	CNS infection	Vancomycin	0

BSI: bloodstream infection; CNS: central nervous system; IE: infective endocarditis NR: not reported; PD: peritoneal dialysis; SSTI: skin and soft tissue infection; VP: ventriculoperitoneal.

**Table 2:** Characteristics of patients with *Brevibacterium* species infection.

Characteristic	All Patients (*n* = 42) *	Bacteremia (*n* = 24) *^,#^	Peritoneal-Dialysis Peritonitis (*n* = 7) *
Age, years, median (IQR)	48 (28–63.5)	46 (19–62)	63 (37–72)
Male sex, *n* (%)	23/40 (57.5)	11/22 (50)	4 (57.1)
Predisposing factors			
Central venous catheter, *n* (%)	17/41 (41.5)	17/23 (73.9)	0 (0)
Recent surgery, *n* (%)	6/36 (16.7)	2/20 (10)	0/5 (0)
End-stage renal disease on peritoneal dialysis, *n* (%)	7 (16.7)	0/23 (0)	7 (100)
Currently on chemotherapy, *n* (%)	7/40 (17.5)	7/22 (31.8)	0 (0)
Neutropenia, *n* (%)	6/39 (15.4)	6/22 (27.3)	0 (0)
Hematological malignancy, *n* (%)	6/40 (15)	6/23 (26.1)	0 (0)
Solid malignancy, *n* (%)	4/40 (10)	3/23 (13)	0 (0)
HIV-positive, *n* (%)	3/40 (7.5)	2/23 (8.7)	0 (0)
Organ transplantation, *n* (%)	2/41 (4.9)	2/23 (8.7)	0 (0)
IVDU, *n* (%)	1/41 (2.4)	0 (0)	0 (0)
Clinical characteristics			
Fever, *n* (%)	29 (69.1)	22 (91.7)	4 (57.1)
Sepsis, *n* (%)	9/41 (22)	8/23 (34.8)	0 (0)
Species			
*B. casei*, *n* (%)	20 (47.6)	12 (50)	3 (42.9)
*B. epidermidis*, *n* (%)	3 (7.1)	2 (8.3)	0 (0)
*B. otitidis*, *n* (%)	3 (7.1)	1 (4.2)	1 (14.3)
*B. massiliense*, *n* (%)	2 (4.8)	1 (4.2)	0 (0)
*B. iadinum*, *n* (%)	1 (2.4)	0 (0)	1 (14.3)
*B. paicivorans*, *n* (%)	1 (2.4)	1 (4.2)	0 (0)
*B. luteolum*, *n* (%)	1 (2.4)	1 (4.2)	0 (0)
*B. sanguinis*, *n* (%)	1 (2.4)	1 (4)	0 (0)
*Brevibacterium* spp., *n* (%)	10 (23.8)	5 (20.8)	2 (28.6)
Treatment			
Vancomycin, *n* (%)	23/40 (57.5)	13/23 (56.5)	4 (57.1)
Cephalosporin, *n* (%)	9/40 (22.5)	1/23 (4.3)	3 (42.9)
Aminoglycoside, *n* (%)	8/40 (20)	5/23 (21.7)	2 (28.6)
Quinolone, *n* (%)	6/40 (15)	4/23 (17.4)	1 (14.3)
Teicoplanin, *n* (%)	4/40 (10)	4/23 (17.4)	0 (0)
Carbapenem, *n* (%)	3/40 (7.5)	3/23 (13)	0 (0)
Aminopenicillin, *n* (%)	3/40 (7.5)	1/23 (4.3)	0 (0)
Antipseudomonal penicillin, *n* (%)	2/40 (5)	2/23 (8.7)	0 (0)
Daptomycin, *n* (%)	2/40 (5)	0/23 (0)	0 (0)
Macrolide, *n* (%)	2/40 (5)	2/23 (8.7)	0 (0)
Linezolid, *n* (%)	1/40 (2.5)	1/23 (4.3)	0 (0)
Antistaphylococcal penicillin, *n* (%)	1/40 (2.5)	0/23 (0)	0 (0)
Rifampicin, *n* (%)	1/40 (2.5)	0/23 (0)	0 (0)
Tetracycline, *n* (%)	1/40 (2.5)	1/23 (4.3)	0 (0)
Surgical management, *n* (%)	10/41 (24.4)	2 (8.3)	2 (28.6)
Central venous catheter removal, *n* (%)	9/17 (52.9)	9/17 (52.9)	2 (28.6)
Treatment duration, weeks, median (IQR)	19.5 (14–35)	14 (10–23.5)	28 (17.5–31.5)
Outcomes			
Deaths due to infection, n (%)	3/39 (7.7)	2/23 (8.7)	0/6 (0)
Deaths overall, n (%)	4/39 (10.3)	3/23 (13)	0/6 (0)

IQR: interquartile range; IVDU: intravenous drug use; *: data are among the number of patients mentioned on top unless otherwise described; ^#^: cases of bacteremia include the cases with infective endocarditis.

**Table 3:** Antimicrobial resistance rates of *Brevibacterium* spp.

Antimicrobial Agent	Resistance (%) *
Trimethoprim/sulfamethoxazole	8/11 (72.7)
Clindamycin	9/13 (69.2)
Penicillin	11/22 (50)
Aminopenicillin	6/13 (46.2)
Cephalosporin	8/23 (34.8)
Aminopenicillin/inhibitor	2/6 (33.3)
Macrolides	3/22 (13.6)
Quinolones	3/27 (11.1)
Aminoglycosides	2/23 (8.7)
Vancomycin	3/37 (8.1)
Tetracycline	1/19 (5.3)
Carbapenems	0/13 (0)

*: data show number of strains resistant to the respective antimicrobial divided by the number of strains with corresponding data. The rest of the cases did not have available data regarding antimicrobial resistance for the respective antimicrobial agent.

**Table 4:** Characteristics of patients with *Brevibacterium* species infection in regard to the outcome.

Characteristic	Survived (*n* = 36) *	Died (*n* = 4) *
Age, years, median (IQR)	44.5 (20.5–61.5)	66 (45.8–90)
Male sex, *n* (%)	20/35 (57.14)	1 (25)
Predisposing factors		
Central venous catheter, *n* (%)	14/35 (40)	2 (50)
Recent surgery, *n* (%)	6/33 (18.2)	0/3 (0)
End-stage renal disease on peritoneal dialysis, *n* (%)	6/35 (17.1)	0 (0)
Currently on chemotherapy, *n* (%)	7/35 (20)	0 (0)
Neutropenia, *n* (%)	5/33 (15.2)	0 (0)
Hematological malignancy, *n* (%)	6/35 (17.1)	0/3 (0)
Solid malignancy, *n* (%)	3/35 (8.6)	0/3 (0)
Organ transplantation, *n* (%)	2 (5.6)	0 (0)
Microbiology		
*B. casei*, *n* (%)	17 (47.2)	2 (50)
*B. epidermidis*, *n* (%)	3 (8.3)	0 (0)
*B. otitidis*, *n* (%)	3 (8.3)	0 (0)
*B. massiliense*, *n* (%)	1 (2.8)	0 (0)
*B. iadinum*, *n* (%)	1 (2.8)	0 (0)
*B. paicivorans*, *n* (%)	1 (2.8)	1 (25)
*B. luteolum*, *n* (%)	1 (2.8)	0 (0)
*B. sanguinis*, *n* (%)	0 (0)	1 (25)
*Brevibacterium* spp., *n* (%)	9 (25)	0 (0)
Type of infection		
Bacteremia, *n* (%)	21 (58.3)	3 (75)
Infective endocarditis, *n* (%)	2 (5.6)	1 (25)
Peritoneal dialysis-associated peritonitis, *n* (%)	6 (16.7)	0 (0)
Osteoarticular infection, *n* (%)	2 (5.6)	0 (0)
Central nervous system infection, *n* (%)	4 (11.1)	0 (0)
Skin and soft tissue infection, *n* (%)	2 (5.6)	0 (0)
Endophthalmitis, *n* (%)	2 (5.6)	0 (0)
Ventriculoperitoneal schunt infection	1 (2.8)	0 (0)
Clinical characteristics		
Fever, *n* (%)	25 (69.4)	3 (75)
Sepsis, *n* (%)	7 (19.4)	2 (50)
Treatment		
Vancomycin, *n* (%)	19 (52.8)	3 (75)
Cephalosporin, *n* (%)	8 (22.2)	0 (0)
Aminoglycoside, *n* (%)	7 (19.4)	0 (0)
Quinolone, *n* (%)	6 (16.7)	0 (0)
Teicoplanin, *n* (%)	4 (11.1)	1 (25)
Carbapenem, *n* (%)	3 (8.3)	1 (25)
Aminopenicillin, *n* (%)	3 (8.3)	0 (0)
Antipseudomonal penicillin, *n* (%)	2 (5.6)	0 (0)
Daptomycin, *n* (%)	2 (5.6)	0 (0)
Macrolide, *n* (%)	2 (5.6)	0 (0)
Linezolid, *n* (%)	1 (2.8)	0 (0)
Antistaphylococcal penicillin, *n* (%)	1 (2.8)	0 (0)
Rifampicin, *n* (%)	1 (2.8)	0 (0)
Tetracycline, *n* (%)	1 (2.8)	0 (0)
Surgical management, *n* (%)	9 (25)	1 (25)
Central venous catheter removal, *n* (%)	8/15 (53.3)	0/2 (0)

IQR: interquartile range; *: data are among the number of patients mentioned on top unless otherwise described.

## Data Availability

No new data were created or analyzed in this study.

## Supplementary Materials

The following supporting information can be downloaded at: https://www.mdpi.com/article/10.3390/microorganisms13051097/s1, File S1: Datasheet used for data extraction.
